# Atrial fibrillation is linked to increased left ventricular fibrosis and inflammation measured by CMR with prognostic implications

**DOI:** 10.1007/s10554-026-03620-0

**Published:** 2026-02-18

**Authors:** Julia M. Treiber, J. Sebastian Wolter, Sören J. Backhaus, Luise von Haugwitz, Thomas Neumann, Malte Kuniss, Jörg Yogarajah, Valentina O. Puentmann, Eike Nagel, Till Keller, Samuel Sossalla, Andreas Rolf

**Affiliations:** 1 Department of Cardiology, Kerckhoff Heart Center, Bad Nauheim, Germany; 2 University of Giessen, Medical Clinic I, Giessen, Germany; 3 Partner site Rhine-Main, German Center for Cardiovascular Research (DZHK), Frankfurt am Main (Main site), Germany; 4 Institute of Experimental and Translational Cardiovascular Imaging, University Hospital Frankfurt, Frankfurt am Main, Germany; 5 Department of Cardiology, Kerckhoff Heart and Thorax Center, Benekestrasse 2-8, 61231 Bad Nauheim, Germany; 6 Justus-Liebig-Universität Gießen (JLU), Gießen, Hesse Germany

**Keywords:** Atrial fibrillation, T1-mapping, Fibrosis, Inflammation, Left ventricular dysfunction, Prognosis

## Abstract

Approximately 30% of patients with atrial fibrillation (AF) develop heart failure (HF), but the underlying mechanisms and their impact on the left ventricle (LV) remain unclear. Fibrosis and inflammation are suspected contributors. This study aimed to investigate LV tissue characteristics in patients with and without AF using cardiac magnetic resonance imaging (CMR). Patients from a single-center CMR registry were categorized by AF status, adjudicated by two blinded investigators. LV fibrosis was assessed by native T1 and extracellular volume (ECV), while T2 indicated inflammation. Results were adjusted for LV ejection fraction (LVEF), end-systolic volume index (ESVi), and late gadolinium enhancement mass (LGEmass). Prognostic value was tested with multivariable Cox regression, using all-cause mortality or HF hospitalization at one year as the endpoint. Of 2,879 patients with one-year follow-up, 590 had AF. They showed lower LVEF and higher ESVi and LGEmass. After adjustment, AF was linked to higher T1 (*p* = 0.006), T2 (*p* = 0.01), and ECV (*p* < 0.001). Eighty-five patients reached the endpoint. In multivariable analysis, only T1 independently predicted outcome. Kaplan-Meier analysis showed patients with T1 above the median (1125 ms) had worse outcomes than those below, even without AF. The combination of AF and elevated T1 carried the poorest prognosis (*p* = 0.001). AF is closely associated with LV fibrosis and inflammation, with T1 emerging as an independent predictor of outcome. These findings suggest a mechanistic pathway with important prognostic implications. Prospective studies are warranted to confirm these associations and explore their role in patient stratification and management.

**Graphical Abstract:**

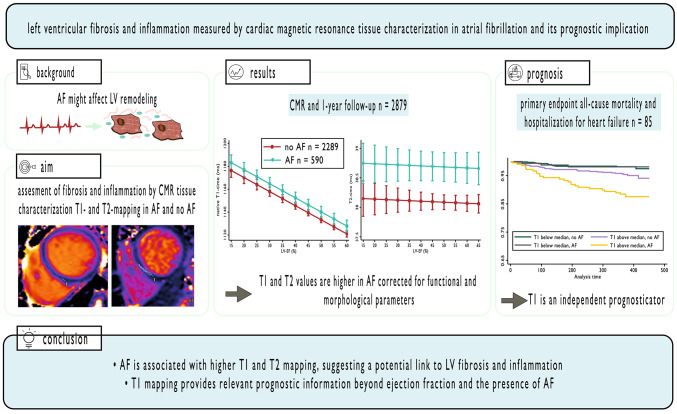

## Introduction

Atrial fibrillation (AF) may trigger left ventricular systolic dysfunction (LVSD) or exacerbate pre-existing heart failure (HF). The underlying pathomechanism has been extensively discussed and appears to involve a combination of morphological left ventricular (LV) changes, cellular and extracellular remodeling, neurohumoral adaptive mechanisms, disturbed intrinsic myocardial contractile function and pro-apoptotic cascades [1–4]. Recent data suggest that LVSD is primarily triggered by arrhythmic stimulation rather than solely by tachycardic conduction [3]. This is further supported by clinical data demonstrating that catheter-based rhythm control is superior to rate control alone in patients with HF [5, 6]. However, the significance of AF for the ventricular microarchitecture remains a subject of ongoing scientific investigation. Since LVSD in early stages of arrhythmia induced LVSD is potentially reversible [7], identifying features like myocardial fibrosis and inflammation, which might mediate the aforementioned tissue alterations, are of paramount significance.

Cardiac magnetic resonance imaging (CMR) provides the unique opportunity to quantitively assess fibrosis and inflammation by T1 and T2 mapping [8–10]. Both T1 and T2 mapping are reliable tools that can yield prognostic data in various cardiovascular diseases with reduced and preserved LV ejection fraction (LV-EF) [8, 11]. Some studies indicate differences in T1 mapping parameters for AF patients compared to healthy controls that reflect its prognostic value for rhythm and LV-EF restoration [12–14]. Even though inflammation is known to be a substrate and cause of AF, to the best of our knowledge there are no existing data regarding T2 mapping as a marker of inflammation in AF [15].

The diagnosis of arrhythmia induced LVSD requires exclusion of other primary causes and can only be established retrospectively. Identifying typical changes in tissue characteristics via CMR could be crucial in the diagnostic process. Therefore, the aim of the following study was to evaluate the influence of AF on CMR-derived tissue characteristics indicative of fibrosis and inflammation and to assess their prognostic implications in AF patients.

## Methods

### Study population

The present study population was drawn from a prospective CMR/biobank (BioCVI) all-comers registry of patients who underwent clinically indicated CMR at a single center (Kerckhoff Heart and Thorax Center, Bad Nauheim, Germany) between April 2017 and May 2023. The registry database contains the answers to a standardized questionnaire including information about symptoms, clinical and family history, and medication. Routine follow-up questionnaires were administered one year after enrolment by telephone or in writing. Clinical indications for CMR were obtained by assessment of myocardial function, ischemia testing, viability testing, myocarditis, and differentiation of cardiomyopathy. All patients gave their written informed consent. The registry was approved by the ethics committee of the University of Giessen.

### Definition of AF

The presence of AF was adjudicated after review of available electrocardiograms or was based on general practitioner records, previous hospital reports, or self-reported AF with concomitant AF-related medication (e.g. anticoagulants, flecainide, or amiodarone).

### CMR acquisitions

CMR was performed on a 3.0 Tesla scanner (Skyra, Siemens Healthineers, Erlangen, Germany) in the head-first supine position with an 18-array coil. All CMR scan protocols followed the Society of Cardiovascular Magnetic Resonance recommendations [16]. Routine scans included native T1 and T2 mapping, functional assessment in 3 standard long-axis and short-axis stacks, LGE, and post-contrast medium T1 mapping. Perfusion scans were added if clinically necessary. Image analysis was performed on a commercially available workstation (CVI42, Calgary, Canada) by two experienced examiners (AR 18 years and JT 8 years).

### CINE imaging

Retrograde electrocardiogram gated steady-state free precession cine images (typical scan parameters: echo time 1.38 ms, repetition time 3.15 ms, flip angle 50°, bandwidth 962 Hz/px, field-of-view 380 mm, voxel size 1.8 × 1.8 mm, slice thickness 8 mm, interslice gap 2 mm, temporal resolution 30 ms) were used routinely for functional analysis. The myocardium was covered from base to apex with 11–15 short-axis and with 3 standardized long-axis (2-, 3-, and 4-chamber view) stacks. Two-dimensional LV strain was assessed by feature tracking in all long-axis scans for global longitudinal strain (GLS) and in additional short-axis scans for global circumferential strain (GCS). Contour motion and strain curves were checked for plausibility.

### Native T1 and T2 mapping

T1 and T2 maps were generated in 3 short-axis scans following 5 into 3 planning. Native T1 mapping was performed by MOdified Look Locker Inversion (MOLLI) recovery sequences 3(2)3(2)5) (typical scan parameters: echo time 1.14 ms, repetition time 3.1 ms, bandwidth 108 Hz/px, field-of-view 350 mm, voxel size 1.4 × 1.4 × 8.0 mm, slice thickness 8 mm, adiabatic inversion pulse, 11 inversion times) and electrocardiogram gated antegrade steady state free precession single-shot readout with 50° flip angle. T2 maps were generated using electrocardiogram gated antegrade T2 prep steady state free precession sequences generated with the breath-hold technique (typical scan parameters: echo time 1.34 ms, repetition time 4.2 ms, flip angle 12°, voxel size 1.8 × 1.8 mm, slice thickness 8 mm, and T2 prep with 0, 30, and 55 ms). Native global T1 and T2 times were determined in the midventricular or basal septum. A region-of-interest at least 2 voxels wide was placed in the center of the septum as recommended by the ConSept method in an automatically generated parametric T1 and T2 map, and mean values were calculated [17]. To avoid partial volume effects of the blood pool, the accuracy of motion correction of the native magnitude images was carefully monitored. ECV was calculated using the previously published formula based on pre- and post-contrast T1 values and hematocrit [18].

### Late gadolinium enhancement

LGE inversion recovery-segmented gradient echo sequences (typical scan parameters: echo time 1.97 ms, repetition time 3.5 ms, flip angle 20°, bandwidth 289 Hz/px, field-of-view 370 mm, voxel size 1.3 × 1.3 × 8.0 mm, and slice thickness 8 mm) were obtained 10 to 15 min after intravenous administration of gadolinium-dota (Dotarem^®^, Guerbet, Villepinte, France; 0.15 mmol/kg bodyweight). Nine to eleven short-axis and 3 long-axis scans were positioned identically to cine imaging. For quantitative assessment a deviation of 5 standard deviations from a reference region was defined as pathological, and LGE mass and relative LGE mass were calculated automatically by the software.

### Study endpoints

Study endpoints were defined according to the 2014 ACC/AHA Key Data Elements and Definitions for Cardiovascular Endpoint Events in Clinical Trials [19]. The primary endpoint was defined as a combination of all-cause mortality and hospitalization for HF after one year. As secondary endpoint the combination of major adverse cardiac events (MACE), all-cause mortality, non-fatal myocardial infarction, non-fatal stroke/transient ischemic attack, and hospitalization for unstable angina pectoris was defined. All recorded endpoints were adjudicated in an endpoint conference.

### Statistics

Normally distributed metric parameters are presented as mean ± standard deviation and in cases of non-normal distribution as median and interquartile range. Categorial variables are presented as absolute frequencies and percentages. Normality of the data was determined by the Shapiro-Wilk test. Groups were compared using a t-test in cases with normal distribution and Wilcoxon signed-rank test for non-normally distributed dependent data. To evaluate the effect of AF on CMR tissue characteristics, patients were divided in AF and non-AF groups and an ANCOVA was performed controlling for LV-EF, LV-ESVi, and LGE mass.

Univariate and multivariable Cox proportional hazards regression analyses were performed to evaluate the prognostic value of independent variables that included volumetric CMR parameters, tissue characterization markers, the presence of atrial fibrillation, and baseline characteristics. Initially, univariate Cox regression analyses were conducted for each independent variable to identify those significantly associated with the outcome. The variables with the highest Wald statistic values in the univariate analyses were selected for inclusion in the multivariable Cox regression model to assess their independent prognostic value. Multicollinearity among covariates included in the multivariable Cox regression model was assessed using variance inflation factors (VIF). The proportional hazards assumption was tested and confirmed for all models. Results are reported as hazard ratios (HR) with 95% confidence intervals (CI). Survival analysis was carried out using the Kaplan-Meier method. A p-value of less than 0.05 was considered statistically significant. Differences in survival curves were assessed using the log-rank test. All tests were computed using Stata software (Stata Corp, College Station, Texas, USA).

## Results

### Baseline characteristics

From April 2017 to May 2023, a total of 2.879 patients with clinical indication for CMR were enrolled in the BioCVI registry and completed the one-year follow-up. The majority were male (74.4%), 590 had a history of AF. Patients with AF were older, more frequently male, and had a higher prevalence of cardiovascular risk factors including hypertension, diabetes, and dyslipidemia, as well as higher NT-proBNP levels, compared to patients without AF (Table 1). Those with an AF history had more frequently pathological CMR findings (Table 1).

**Table 1:** Baseline characteristics

Baseline characteristic	No atrial fibrillation *n* = 2,289	Atrial fibrillation *n* = 590	*p*-value
Age, y	57 ± 16	68 ± 11	<0.001
Male sex	1466 (64)	439 (74)	<0.001
Body mass index, kg/m^2^	27 ± 6	28 ± 5	0.005
NT-pro-BNP, pg/ml	1064 ± 3311	1999 ± 3600	<0.001
Troponin T, pg/ml	58 ± 307	54 ± 184	0.745
Glomerular filtration rate, ml/min/1,73 m^2^	95 ± 31	78 ± 31	<0.001
Hypertension	1327 (58)	449 (76)	<0.001
Dyslipidemia	1032 (45)	333 (56)	<0.001
Diabetes	398 (17)	134 (23)	0.003
Smoker	522 (23)	84 (14)	<0.001
Past smoker	735 (32)	249 (42)	<0.001
*Diagnosis after CMR*			
Normal findings	587 (26)	100 (17)	<0.001
Chronic coronary syndrome	471 (21)	117 (20)	
Valvular heart disease	7 (0.3)	1 (0.2)	
Ischemic cardiomyopathy	393 (17)	129 (22)	
Dilated cardiomyopathy	312 (14)	119 (20)	
Hypertrophic cardiomyopathy	42 (2)	14 (2)	
Other (e.g. cardiac mass)	20 (1)	16 (3)	
Hypertensive heart disease	37 (2)	2 (0.3)	
Storage disease	8 (0.3)	9 (1.5)	
Pulmonary hypertension/right heart failure	20 (1)	4 (0.7)	
Myocardial inflammation	135 (6)	33 (6)	
Acute myocardial injury	20 (1)	5 (1)	
Control cohort	64 (3)	0	

Data represent mean ± standard deviation or n (%).NT-pro-BNP = N-terminal fragment of brain natriuretic peptide

### CMR parameters

**Fig. 1:**
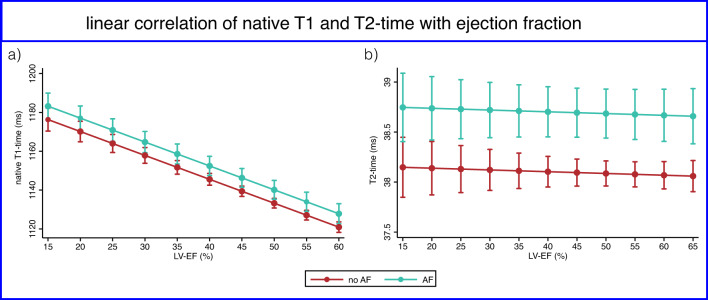
linear correlation of native T1-mapping (**a**) and T2-mapping (**b**) with left ventricular Ejection fraction. LV-EF = left ventricular ejection fraction, AF = atrial fibrillation

AF patients had significantly lower LV-EF and impaired morphological CMR parameters (Table 2). Specifically, AF patients exhibited higher LV end-systolic volume index, impaired global longitudinal and circumferential strain, and a greater relative LGE burden. Native T1 and T2 mapping parameters as well as ECV were higher in AF patients. This effect remained significant after adjusting for functional (LV-EF), morphological (ESVi), and structural (LGE mass) parameters. Plotted against LV-EF, native T1 and T2 times increased with decreasing LV-EF; however, at each level of LV-EF, T1 and T2 were always higher in AF patients (Fig. 1). The correlation between native T1 and LV-EF was statistically significant with a modest goodness of fit (R² = 0.094, *p* < 0.001), whereas T2 showed a weak, non-significant trend towards higher values with decreasing LV-EF (R² = 0.001, *p* = 0.082).

**Table 2:** Baseline CMR parameters

CMR parameter	No atrial fibrillation *n* = 2,289	Atrial fibrillation *n* = 590	*p*-value
LV-EF [%]	52 ± 15	48 ± 16	<0.001
LV-ESVi [ml/m^2^]	46 ± 31	50 ± 31	0.007
Myocardial mass index [g/m^2^]	56 ± 20	57 ± 19	0.059
GLS [%]	−16 ± 5	−13 ± 5	<0.001
GCS [%]	−17 ± 5	−15 ± 6	<0.001
Relative LGE mass [%]	4 ± 6	5 ± 8	<0.001
T1 [ms]	1131 ± 61	1143 ± 69	<0.001
T2 [ms]	38.7 ± 3	39.3 ± 3	<0.001
ECV	0.26 ± 0.09	0.28 ± 0.09	0.003
*Tissue characteristics controlled for LV-EF, LV-ESVi, and LGE mass*			
T1, ms	1130 ± 65	1138 ± 128	0.0063
ECV	0.262 ± 0.1	0.272 ± 0.2	0.0005
T2, ms	38.7 ± 4	39.2 ± 7	0.0099

Data represent mean ± standard deviation. ECV = extracellular volume fraction, LV-EF = left ventricular ejection fraction, LV-ESVi = end-systolic volume index, GCS = global circumferential strain, GLS = global longitudinal strain, LGE = late gadolinium enhancement

### Outcome analysis

After one year follow-up 85 patients reached the primary endpoint (all-cause mortality, *n* = 39; hospitalization for HF, *n* = 46) and 155 reached the secondary endpoint (myocardial infarction, *n* = 21; stroke, *n* = 20; transient ischemic attack, *n* = 11; hospitalization for unstable angina pectoris, *n* = 64).

In a univariate analysis incorporating morphological and functional parameters as well as tissue characterization variables, all variables including the presence of AF except for sex and troponin were found to be significant predictors of the primary endpoint. However, in a multivariable model only T1 remained an independent predictor. Multicollinearity among variables included in the multivariable Cox regression model revealed no relevant multicollinearity (mean VIF 1.57, maximum VIF 2.21 for GLS). For the secondary endpoint, ECV was also a significant predictor but lost independence when adjusted for age and NT-proBNP. (Tables 3 and 4).

**Table 3:** Cox regression for the primary endpoint all-cause mortality and HF hospitalization, univariate and multivariable analyses

	Univariate analysis					Multivariable analysis			
	HR	LL	UL	Wald	*p*-value	HR	LL	UL	*p*-value
T1	1.01	1.007	1.013	50.3	0.001	1.008	1.004	1.011	<0.001
ECV	9.731	2.358	40.168	9.9	0.002				
Relative LGE mass	1.047	1.022	1.072	13.7	<0.001				
T2	1.151	1.070	1.239	14.1	<0.001				
LV-EF	0.960	0.948	0.973	37.1	<0.001				
GCS	1.132	1.087	1.180	34.9	<0.001				
GLS	1.174	1.124	1.226	51.6	<0.001	1.073	1.000	1.152	0.052
LV-EDVi	1.013	1.009	1.018	33.5	<0.001				
LV-ESVi	1.014	1.010	1.019	46.6	<0.001	1.007	1.000	1.014	0.065
Age	1.032	1.014	1.050	12.1	<0.001	1.018	1.000	1.039	0.086
Male sex	0.702	0.421	1.169	1.9	0.174				
NT-pro-BNP	1.0	1.0	1.0001	31.9	<0.001	1.000	1.000	1.000	0.736
Troponin T	1.0	0.999	1.001	0.2	0.660				
eGFR	0.981	0.973	0.989	22.9	<0.001				
AF	2.455	1.548	3.893	14.6	<0.001	1.613	0.918	2.834	0.097

AF = atrial fibrillation ECV = extracellular volume fraction, LV-EDVi = left ventricular end-diastolic volume index, LV-EF = left ventricular ejection fraction, LV-ESVi = left ventricular end-systolic volume index, GCS = global circumferential strain, GLS = global longitudinal strain, HR = hazard ratio, LL = lower limit, LGE = late gadolinium enhancement, UL = upper limit, NT-pro-BNP = N-terminal fragment of brain natriuretic peptide, eGFR = estimated glomerular filtration rate

**Table 4:** Cox regression for the secondary endpoint MACE, univariate and multivariable analyses

	Univariate analysis					Multivariable analysis			
	HR	LL	UL	Wald	*p*-value	HR	LL	UL	*p*-value
Native T1	1.004	1.001	1.007	7.7	0.006				
ECV	11.108	3.169	38.935	14.2	<0.001	5.685	1.082	29.867	0.04
relative LGE mass	1.028	1.005	1.0523	5.5	0.019				
T2	1.051	0.985	1.122	2.3	0.132				
LV-EF	0.993	0.982	1.005	1.2	0.276				
GCS	1.036	1.000	1.073	3.8	0.051				
GLS	1.075	1.034	1.116	13.6	<0.001	0.989	0.941	1.039	0.656
LV-EDVi	0.999	0.993	1.005	0.1	0.782				
LV-ESVi	1.001	0.995	1.007	0.1	0.70				
Age	1.056	1.038	1.074	39.3	<0.001	1.046	1.024	1.068	<0.001
Male sex	0.768	0.500	1.179	1.5	0.228				
NT-pro-BNP	1.000	1.000	1.000	16.9	<0.001	1.000	1.000	1.000	0.009
Troponin T	1.000	0.999	1.001	0.0	0.958				
eGFR	0.986	0.979	0.993	16.1	<0.001				
AF	2.303	1.551	3.419	17.1	<0.001	1.469	0.882	2.448	0.14

ECV = extracellular volume fraction, EDVi = end-diastolic volume index, LV-EF = ejection fraction, LV-ESVi = end-systolic volume index, GCS = global circumferential strain, GLS = global longitudinal strain, HR = hazard ratio, LL = lower limit, LV = left ventricular, rLGEmass = relative late gadolinium enhancement mass, UL = upper limit

**Fig. 2:**
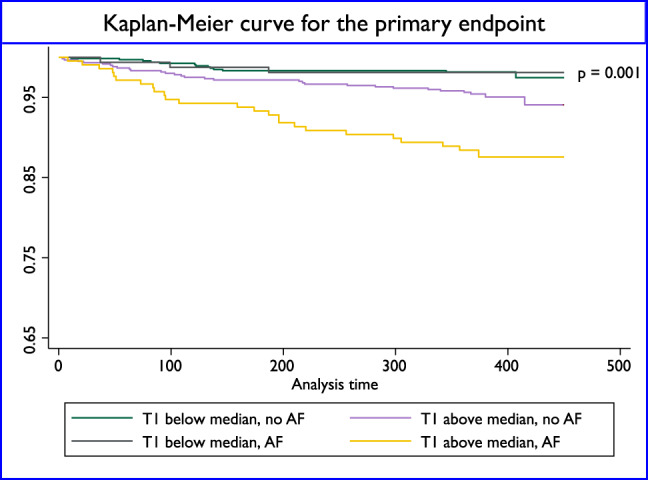
Kaplan-Meier survival estimates for the presence or absence of atrial fibrillation and the median native T1-time of 1125 ms. AF = atrial fibrillation

The Kaplan-Meier curve analysis demonstrated that patients with T1 values above the median of 1125 ms, T1 time had a significantly poorer outcome compared to those with T1 values below the median, even in the absence of AF (Fig. 2). However, if AF was present, patients with T1 times above the median fared even worse (*p* = 0.001).

## Discussion

The data from our registry suggest an association between AF and ventricular remodeling and inflammation reflected by CMR T1 and T2 mapping. Our key findings are:

1. Even after adjusting for morphological and functional parameters as well as structural changes in LGE, patients with AF exhibited higher T1 and T2 mapping values than those without.
2. T1 is the sole independent predictor of the primary endpoint all-cause mortality when considering the covariates AF and GLS.
3. Patients with both AF and elevated T1 showed the worst prognosis with respect to the primary endpoint.

AF is both a consequence and a cause of HF and both disorders frequently coexist [20, 21]. Approximately one-third of all patients with AF develop HF following the initial manifestation of AF [21]. This fact suggests that AF affects both atria and ventricles. In the present registry, AF was classified based on medical history, electrocardiographic documentation, and medication records, without systematic differentiation between paroxysmal and persistent AF. If AF causes LVSD, this condition could potentially be reversible with effective rhythm control. Of note, AF subtype, burden, and duration may differentially affect the extent of ventricular remodeling and tissue characterization parameters. However, our data do not allow differentiation between AF as a causal factor or a consequence of ventricular remodeling. Differentiating these patients is challenging but crucial for management and outcomes. Arrhythmia-induced LVSD is only diagnosed retrospectively, and while early identification would be valuable, our findings remain hypothesis-generating and provide associative mechanistic insights rather than proof of causality.

Structured investigations of the effect of AF on LV tissue remodeling are sparse and the data are conflicting. Histopathological examinations of patients with supraventricular rhythm disorders have confirmed structural alterations of the ventricular myocardium even in the absence of abnormalities due to myocarditis or unspecific changes [22, 23]. Further, the extent of ventricular fibrosis appears to be related to the frequency of AF [1, 24, 25]. The CASTLE-AF trial also demonstrated an advantage of rhythm control via catheter ablation over traditional medical therapy with respect to mortality, hospitalization for HF, and improvement in LV-EF, which at least is consistent with an effect of AF on the LV [5]. However, LVSD in AF patients does not seem to be solely due to altered microarchitecture. Pabel et al. found no difference in the amount of fibrosis in patients with and without AF who had preserved LV-EF and aortic valve stenosis [3]. Instead, they were able to demonstrate reduced calcium release from cardiomyocytes that resulted in disturbed excitation-contraction coupling.

CMR tissue characterization offers the potential to detect structural changes of the myocardial microarchitecture completely noninvasively and might add to the understanding of the underlying mechanisms. It is well known that T1 relaxation time is sensitive to myocardial water content and fibrosis, whereas T2 mapping specifically reflects myocardial edema [8, 10, 26, 27]. T1 and T2 and, with the addition of post-contrast T1 and hematocrit, ECV are therefore also considered as physical biomarkers for inflammation and fibrosis. We found a continuous increase in native T1 time with LV-EF reduction in both AF and no AF patients. However, AF patients showed higher T1 across all LV-EF ranges, also when corrected for functional (LV-EF), morphological (LV-ESVi), and structural (relative LGE mass) covariates. While these observations do not allow for a distinction between AF-induced remodeling and remodeling-induced AF, they suggest a link between ventricular fibrosis and AF.

Shortened post-contrast T1 mapping in AF, particularly in persistent AF, has been reported [14, 24]. However, distinguishing between actual fibrotic changes and technical limitations due to AF can be challenging. Ling et al. found lower post-contrast T1 in patients with LV-EF recovery post-ablation, while Montgomery et al. observed this only in persistent AF(13,15). Therefore, they hypothesized that the observed T1 changes could also be partially due to technical limitations in performing T1 mapping in patients with AF. Zhao et al. demonstrated that MOLLI T1 mapping remains reliable in AF, especially when heart rate-dependent systolic triggering was used [13]. Consistent with our findings, they found elevated T1 and ECV in AF patients compared to those without AF.

Our data convincingly demonstrate the prognostic power of T1 mapping independent of AF history or LVSD regarding the primary endpoint all-cause mortality and hospitalization for HF. Predictably, patients with elevated T1 and evidence of AF had the worst prognosis. Several studies have validated that CMR tissue characterization parameters can predict the success of AF ablation therapy. Most studies focused on the presence of late enhancement. The absence of LGE appears to favor the improvement of LV-EF [28]. Further the CAMERA-MRI study showed that patients without evidence of fibrosis on LGE particularly benefit from ablation-mediated rhythm control compared to medication-based rate control [29]. Interestingly they used LGE as a sign of ventricular fibrosis, which only appears after fibrotic replacement. Against this background, T1 mapping may provide complementary information beyond LGE by capturing diffuse interstitial fibrosis, which precedes replacement fibrosis detectable by LGE. Therefore, T1 mapping could potentially contribute to identifying AF patients with reversible ventricular remodeling who may derive particular benefit from rhythm-control strategies such as catheter ablation. However, this hypothesis requires validation in prospective interventional studies. The use of T1 mapping would even have the advantage of quantifying fibrosis burden and could potentially underscore the results further. In 103 patients with preprocedural CMR, McLellan et al. found a lower post-contrast T1 time in patients with AF recurrence after catheter ablation [30]. In a retrospective analysis of 32 patients, Azuma et al. determined that ECV has an incremental value over LGE for LV-EF improvement of > 10% [31]. These findings highlight the prognostic potential of T1 mapping for a better understanding of the reversibility of arrhythmia induced LVSD.

To the best of our knowledge, this is the first study that investigates differences in T2 mapping in patients with and without AF. Similar to T1, T2 shows a continuous increase at progressively lower LV-EF and is significantly elevated in AF patients when corrected for baseline LV-ESVi, LV-EF, and LGE mass. Clinically apparent as well as subclinical inflammation induces atrial remodeling [15]. However, elevated T2 values should be interpreted cautiously, as they may reflect increased myocardial water content related to inflammation, hemodynamic stress, or volume overload rather than direct evidence of inflammatory myocardial injury. Consequently, our data cannot establish whether AF promotes myocardial inflammation or whether inflammatory processes predispose to AF. Proinflammatory extracardiac processes such as obesity, as well as inflammation directly induced by cardiac surgery or ablation, promote AF [32, 33]. The reduction of AF during anti-inflammatory therapy following cardiac surgery suggests an AF-promoting effect of inflammation [15]. Studies focused on C-reactive protein (CRP) levels as a marker of global inflammatory processes confirmed a correlation of CRP and the evolution of AF [32]. However, CRP is merely an indirect marker of inflammation, and its serum levels can be influenced by other factors. T2 mapping provides the opportunity to quantify myocardial edema and inflammation noninvasively [10]. Our finding of elevated T2 suggests that mild inflammation affecting the myocardium is associated with AF. However, our data cannot differentiate between the possibilities that AF triggers inflammation or that AF is caused by inflammation. Accordingly, T2 mapping should be interpreted as a non-specific marker of myocardial edema, and our data do not allow discrimination between inflammation-driven and hemodynamically mediated T2 elevation. This distinction is particularly relevant in AF patients with concomitant ventricular dysfunction and underscores the need for multimodal assessment in future studies.

Our data suggest that T1 and T2 mapping may help identify structural differences in the LV myocardium in AF patients. Due to the observational nature of this study, these findings should be interpreted as associative rather than causal. While this retrospective, hypothesis-generating analysis links diffuse fibrosis and inflammation to ventricular remodeling, it cannot establish causality, requiring further prospective studies. However, given its prognostic value, CMR should gain importance in clinical practice to better identify patients who require closer monitoring and targeted management.

## Limitations

The main limitation of our study is its observational character, preventing differentiation between cause and effect. Our registry only recorded a “history of AF” without distinguishing between AF subtypes, limiting the assessment of AF persistence on CMR characteristics. Additionally, AF duration, frequency, and heart rate may influence the ventricular myocardium. Medication-based AF classification poses challenges, as anticoagulation and antiarrhythmic therapy may be prescribed for other conditions, introducing potential misclassification bias. T1 and T2 mapping also have limitations in tachycardic AF, and despite plausibility checks, signal contamination from partial volume effects cannot be entirely excluded. To minimize this, we adhered to the conSept methodology and validated motion correction in raw data [17].

## Conclusion

Our results indicate that AF is associated with LV fibrosis and inflammation offering significant prognostic insights. CMR T1 and T2 mapping provide additional details on myocardial microarchitecture and prognostic information in AF patients.

## Data Availability

The data underlying this article cannot be shared publicly in order to protect the privacy of individuals who participated in the study. The data will be shared on reasonable request to the corresponding author.

## Abbreviations

AF: atrial fibrillation
CMR: cardiac magnetic resonance imaging
ECV: extracellular volume fraction
HF: heart failure
LGE: late gadolinium enhancement
LV: left ventricle
LV-EF: left ventricular ejection fraction
LV-ESVi: end-systolic volume index
LVSD: left ventricular systolic dysfunction
MACE: major adverse cardiac events
